# COCHRANE REVIEW: CONCLUSIONS ABOUT THE EFFECTS OF ELECTRONIC CIGARETTES REMAIN THE SAME

**Published:** 2017-01

An updated Cochrane Review published today provides an independent, rigorous assessment of the best available evidence to date about electronic cigarettes for quitting smoking.

The conclusions of this updated Review are unchanged since the last review was published two years ago: electronic cigarettesmay help smokers stop their smoking, and the included studies did not find any serious side effects associated with their use for up to two years.

Many studies are now underway which may help us understand more about their effects in the future.

The first Cochrane Review, published in the Cochrane Library in December 2014, showed that electronic cigarettes may be an aid to smokers in stopping their smoking. The updated Review did not find any new randomized controlled trials (RCTs) with long-term outcomes looking at the effectiveness of electronic cigarettes in helping people to stop smoking. However, this is an active area of research, with a large number of ongoing studies that will add to the evidence in the next few years.

Smoking is a significant global health problem. Despite many smokers wanting to stop, they often find it difficult to succeed in the long-term. One of the most effective and widely used strategies to help combat the cravings associated with nicotine addiction is to deliver nicotine by patches and chewing gum.

Electronic cigarettes have been around in some form for a number of years, but over the past few years their popularity has increased significantly, and they have begun to look and feel less like conventional cigarettes. Unlike chewing gum and patches, they mimic the experience of cigarette smoking because they are hand-held and generate a smoke-like vapour when used. They help to recreate similar sensations of smoking without exposing users or others to the smoke from conventional cigarettes, and can be used to provide smokers with nicotine. Though they are used by many smokers, little is still known about how effective they are at helping people stop smoking.

This version of the updated Cochrane Review includes no new RCTs. The original Review included two RCTs involving more than 600 participants, and found that electronic cigarettes containing nicotine may increase the chances of stopping smoking within six to 12 months, compared to using an electronic cigarette without nicotine. The researchers could not determine whether using electronic cigarettes was better than a nicotine patch in helping people stop smoking, because there were not enough people taking part in the study.

This updated Review now includes observational data from an additional 11 studies. Of the studies which measured side effects, none found any serious side effects of using electronic cigarettes for up to two years. The studies showed that throat and mouth irritation are the most commonly reported side effects in the short-to medium-term (up to two years).

The lead author of this Cochrane Review, Jamie Hartmann-Boyce from the Cochrane Tobacco Addiction Group, said, “The randomized evidence on smoking cessation is unchanged since the last version of the Review. We are encouraged to find many studies are now underway, particularly as electronic cigarettes are an evolving technology. Since the last version of the Review, 11 new observational and uncontrolled studies have been published. In terms of quitting, these can’t provide the same information we get from randomized controlled trials, but they contribute further information on the side effects of using electronic cigarettes to quit smoking. None detected any serious side effects, but longer term data are needed.”

*Full citation: Hartmann-Boyce J, McRobbie H, Bullen C, Begh R, Stead LF, Hajek P. Electronic cigarettes for smoking cessation. Cochrane Database of Systematic Reviews 2016, Issue 9. Art. No.: CD010216. DOI: 10.1002/14651858.CD010216.pub3*.

*Copyright © 2016 The Cochrane Collaboration. Published by John Wiley & Sons, Ltd., reproduced with permission*.

